# A Trauma Support App for Young People: Co-design and Usability Study

**DOI:** 10.2196/57789

**Published:** 2025-03-18

**Authors:** Maria Thell, Kerstin Edvardsson, Reem Aljeshy, Kalid Ibrahim, Georgina Warner

**Affiliations:** 1 Child Health and Parenting, Department of Public Health and Caring Science Uppsala University Uppsala Sweden

**Keywords:** co-design, young people, trauma, app development, usability testing

## Abstract

**Background:**

One of the most common reasons young people with mental health issues, such as posttraumatic stress disorder, do not seek help is stigma, which digital support tools could help address. However, there is a lack of trauma support apps specifically designed for young people. Involving the target group in such projects has been shown to produce more engaging and effective results.

**Objective:**

This study aimed to apply a child rights–based participatory approach to develop a trauma support app with young people.

**Methods:**

Seven young people (aged 14-19 years; 3 males and 4 females) with experiences of trauma were recruited as coresearchers. A child rights–based framework guided the working process. The app was developed through a series of Design Studio workshops and home assignments, using the manualized intervention Teaching Recovery Techniques as the foundation for its content. The coresearchers were trained in research methodology and conducted usability testing with other young people (n=11) using the think-aloud method, the System Usability Scale (SUS), and qualitative follow-up questions.

**Results:**

A functional app prototype was developed using a no-code platform, incorporating various trauma symptom management techniques. These techniques covered psychoeducation, normalization, relaxation, and cognitive shifting, presented in multiple formats, including text, audio, and video. The contributions of the coresearchers to the design can be categorized into 3 areas: mechanics (rules and interactions shaping the app’s structure), dynamics (user-visible elements, such as the outcome when pressing a button), and aesthetics (the emotional responses the app aimed to evoke in users during interaction). Beyond influencing basic aesthetics, the coresearchers placed significant emphasis on user experience and the emotional responses the app could evoke. SUS scores ranged from 67.5 to 97.5, with the vast majority exceeding 77.5, indicating good usability. However, usability testing revealed several issues, generally of lower severity. For instance, video content required improvements, such as reducing light flickering in some recordings and adding rewind and subtitle selection options. Notably, the feature for listening to others’ stories was removed to minimize emotional burden, shifting the focus to text formats with more context.

**Conclusions:**

Young people who have experienced trauma can actively participate in the cocreation of a mental health intervention, offering valuable insights into the needs and preferences of their peers. Applying a child rights–based framework to their involvement in a research project supported the fulfillment of the Convention on the Rights of the Child Article 12.

## Introduction

### Background

It is well recognized that one of the main barriers preventing young people from seeking face-to-face help for mental health problems, such as symptoms of posttraumatic stress disorder (PTSD), is stigmatization and embarrassment [1-3]. This includes perceived stigma, fear of stigma, societal stigma, and self-stigma [2], and may result in many young people experiencing PTSD symptoms not receiving the help and support they need. However, these well-known barriers can potentially be overcome through app-based support [3], as mental health apps can engage young people who might not otherwise seek help through traditional routes. Particularly, in the case of PTSD, which differs from other mental illnesses due to its clear cause, young people could receive scalable trauma interventions without delay through digital technology [4]. Despite this, there is a lack of apps specifically developed for young people [5,6].

Teaching Recovery Techniques (TRT) is a manualized group intervention for children aged 8 and older with trauma symptoms [7]. The intervention consists of 5 sessions for children and 2 sessions for parents and caregivers. The content for children and adolescents includes components aligns with trauma-focused cognitive behavioral therapy: psychoeducation, trauma narrative, affective modulation, cognitive coping and processing, in vivo mastery of trauma memories, and future development. TRT helps normalize responses to trauma and provides strategies to manage intrusive thoughts and memories as well as regulate arousal. Participants in a TRT group also work on gradually exposing themselves to thoughts and situations they avoid [7]. TRT has been evaluated in randomized controlled trials with children and young people in several international settings, with an overall positive impact on symptoms of PTSD and depression reported [8]. The initial idea for the current project emerged during a previous participatory study in which TRT was adapted to an online format in response to the COVID-19 pandemic [9]. During the adaptation phase, which involved TRT group leaders, parents, and young people with experience of TRT, the young people suggested consolidating all the techniques into a separate digital format, that is, a mobile app [9].

There has been a growing use of participatory methods with children and young people in the development of health interventions [10]. Previous research indicates that involving the target group in such projects can lead to more engaging and useful outcomes [11,12]. However, when conducting participatory research in the context of PTSD, it is important to recognize that traumatic events create a power imbalance, where one entity holds power over another. An individual’s experience may be shaped by feelings of powerlessness, humiliation, guilt, shame, betrayal, or silencing. In interpersonal interactions, especially in public health research, it is crucial to ensure that these feelings of powerlessness are not replicated or reinforced [13].

It has been suggested that research involving young people as coresearchers should be conducted within a rights-based framework [14]. One argument is that this approach can help address the power imbalance between adults and children. The Convention on the Rights of the Child (CRC) [15] acknowledges the position and disempowerment of children in matters affecting them. Adopting a child rights–based approach as a researcher involves implementing and being accountable to the rights set out in the CRC [16]. For this study, we applied the Lundy model [17] of child and youth participation, including the associated planning checklist for researchers [18]. The Lundy model conceptualizes the two components of Article 12 of the CRC: (1) the right to express views and (2) the right to have those views duly considered. By organizing the 4 elements—space, voice, audience, and influence—in a logical, chronological order, the model helps researchers plan, implement, and monitor projects. Space refers to providing a safe and inclusive environment for children to express their views and ensuring that all children can participate. Voice involves offering appropriate information and facilitating the expression of children’s views. Audience ensures that children’s views are communicated to someone responsible for listening. Influence involves ensuring that children’s views are taken seriously and acted upon where appropriate [17].

### Objective

This study aimed to (1) apply a child rights–based participatory approach to developing a trauma support app with young people who have personal experiences of trauma and (2) conduct usability testing of the app with the young people as coresearchers.

## Methods

### Phase 1: App Development

#### Coresearcher Recruitment

Seven young people were recruited for the project through 3 different channels. Two of them had previously collaborated with the research team on a related TRT research project [9] and were invited to participate again. Additionally, contact was made with a youth-run organization for young people with experience in out-of-home care, which agreed to distribute the recruitment advertisement to its members. Furthermore, a youth center in an area categorized as “vulnerable” by the Swedish Police Authority due to high rates of crime and social exclusion [19] posted the advertisement on its physical noticeboard and social media accounts. Examples of traumatic events and situations, as well as PTSD symptoms, were included in the recruitment materials. Young people who identified with these experiences and enjoyed being creative were encouraged to contact the researchers via email, SMS text message, or WhatsApp (Meta Platforms, Inc.). The advertisement emphasized that participants were not expected to share their personal experiences while working on the project.

Following an initial individual dialogue with all interested young people, 7 chose to participate as coresearchers in the project. The youth coresearcher team consisted of 3 boys and 4 girls, aged 14-19 at the start of the project. They lived in different neighborhoods in 1 large city and 1 medium-sized city in southern Sweden. The older youth attended both theoretical and practical secondary school programs. In accordance with the regulations of the Swedish Work Environment Authority [20], information about the project was provided to the guardians of participants under 18 years of age. Additionally, permission to participate was obtained from the guardians of those under 16 years of age [20]. The coresearchers were remunerated for their time spent on project activities in line with existing recommendations [21].

**Figure 1:**
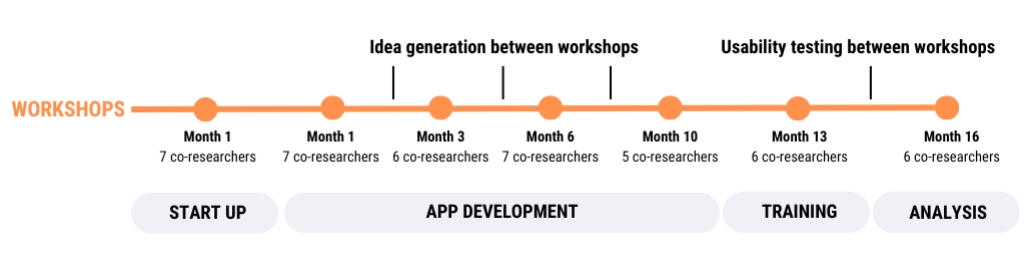
Overview of the Teaching Recovery Techniques app development process with youth co-researchers conducted over 10 months.

Before starting the app development process, a “start-up” session was conducted (Figure 1) to help the team get acquainted through games and other activities. During this session, decisions were made about how to collaborate, including setting common approaches and ground rules to guide the work throughout the project. At the end of the session, an experienced TRT group leader visited to introduce the intervention. As part of this introduction, they guided the group through a popular visual imagery technique called “Safe Inner Place.”

#### App Development Workshops

The app was developed over 4 workshops spanning 10 months (Figure 1), using an adapted version of the Design Studio Workshop method [22]. In addition, the coresearchers individually generated ideas between workshops. To support their independent work, app design prototypes, along with questions and topics for the next workshop, were shared with them. The researcher leading the workshops (MT) collected the individual contributions before each session and presented them to the group for collaborative refinement.

**Figure 2:**
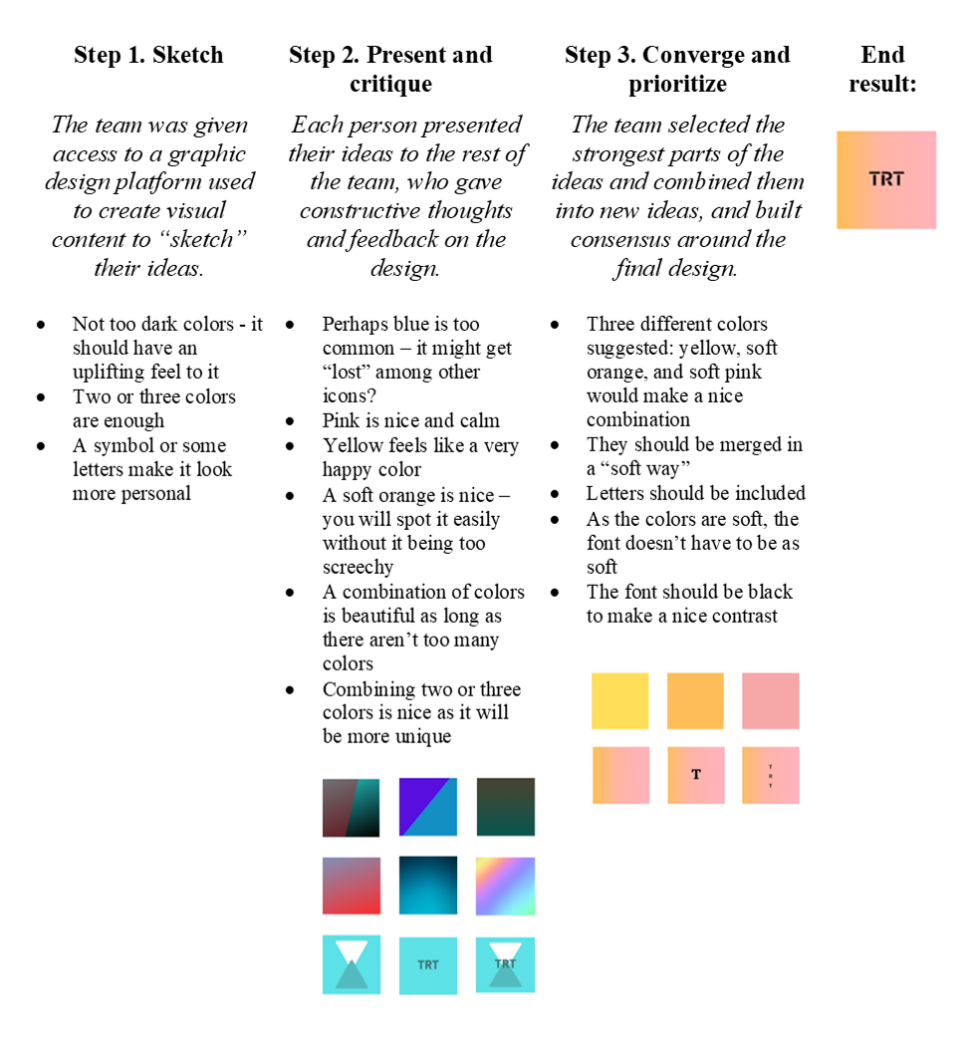
Overview of the Teaching Recovery Techniques (TRT) app logo development process using the Design Studio method.

The first app development workshop aimed to cocreate a logo for the app and pilot the Design Studio Workshop method [22]. The coresearchers were given tablets with internet access and individually explored ideas, first by analyzing their favorite app logos and then by considering a design for a trauma support app. They submitted their ideas to the researcher leading the workshops (MT), who compiled them and projected them onto a screen using a digital noticeboard software tool [23]. Applying the Design Studio method, the team explored colors, fonts, and other design elements. The final logo incorporated the strongest elements from the generated ideas, featuring “soft,” “encouraging,” and “happy” colors while avoiding excessive or overused colors in app logos. The coresearchers selected a font that contrasted with the background and stood out from other popular apps. Figure 2 illustrates the logo development process.

The second workshop focused on prioritizing content and layout for the app using the TRT manual. In the preparatory phase, coresearchers were provided with concise written descriptions of the techniques outlined in the TRT manual. While simplified, these descriptions carefully preserved the original phrasing to maintain the integrity of the intervention logic. Coresearchers were tasked with considering how each technique could be integrated into an app format. To accommodate diverse preferences and abilities, they were encouraged to express their ideas in various ways—whether orally, in writing, or through visual representations such as images or vision boards. Consequently, the initial “sketch” step of the Design Studio method was conducted independently. Upon convening for the workshop, participants engaged in the second step, during which the researcher leading the workshops (MT) presented the collective ideas and facilitated a critique of the proposed concepts. The ideas were then synthesized and prioritized. To discuss app content related to traumatic experiences, case vignettes were used in the workshops, allowing coresearchers to explore different traumatic experiences in a general rather than personal context. Following the workshop, the selected concepts were developed into a prototype of the TRT app using a no-code app-building program [24].

During the third workshop, the coresearchers were presented with an app prototype developed by a research assistant (KE), incorporating their ideas. They provided feedback on both the content and design. For certain techniques, the prototype successfully met their expectations. In other cases, minor adjustments were made in real time during the workshop using the app-building program [24]. For techniques requiring more substantial revisions, the Design Studio cycle was reinstated. This process involved coresearchers initially “sketching” the necessary amendments, followed by presentations and critiques, ultimately converging and prioritizing the changes. Having access to the app-building program during the workshops facilitated this process, as coresearchers could explore the range of functionalities available within the program. Ideas that the coresearchers found valuable but were not feasible to implement at the time were placed in what the team collectively called the “ice box.” This meant that the ideas would be revisited in any future development of the app. One such example was the app’s color scheme, which had been jointly agreed upon. Some coresearchers had also expressed a preference for an alternative, slightly darker color scheme for nighttime use. While the first prototype featured only a single color scheme, the idea of selectable color schemes was considered both valuable and important. Rather than being discarded, it was added to the ice box for future consideration.

Further revisions to the app were made after workshop 3 and presented to the coresearchers for feedback in workshop 4. Some changes, primarily concerning the wording of written content, were informed by discussions among the academic researchers to ensure the integrity of the TRT intervention logic. Any modifications resulting from these discussions were communicated to the coresearchers during workshop 4. As in the previous workshop, coresearchers were invited to provide feedback on the prototype. After a series of minor revisions, they expressed their satisfaction with the product, and a collective decision was made to transition from app development to the usability testing phase of the project.

Overall, attendance at the workshops remained consistently high (Figure 1). One of the 7 coresearchers participated in the entire development process but did not take part in the training and usability testing described below. Other absences were due to illness and were compensated for through individual meetings or conversations with the researcher leading the workshops (MT). Those who were ill also completed the home assignments related to the missed session.

### Phase 2: Usability Testing

#### Coresearcher Training

The usability testing was conducted by the coresearchers, with no academic researchers present in the room during data collection. Given the youth-led nature of the process, training sessions were held before the usability testing began (Figure 1). The training covered both theoretical and practical aspects of research ethics and methodology, enabling the coresearchers to actively lead and carry out the research tasks [25-27].

Careful consideration was given to the safety of study participants. As part of the training, the coresearchers and the academic researcher reflected together on how to recognize when a person might be negatively affected and practiced the necessary actions to take if needed. While training is considered essential for young people conducting research, it is also important to balance the extent of training with maintaining their ability to participate meaningfully [14]. Although the training in this study was based on existing guidelines for research with children [25], it was adapted for young people by condensing the content into 3 sessions held on the same day, with breaks in between. Plans were in place to provide additional sessions if needed, but both the coresearchers and researchers agreed that the initial sessions provided sufficient preparation.

#### Participant Recruitment

The target group for the usability testing consisted of young people aged 15-21 years who spoke and understood Swedish. As the purpose of the usability testing was to assess the intuitiveness of the design and evaluate it with users who had no prior exposure to the app—rather than to determine its efficacy as a trauma support tool—a population sample was recruited instead of a clinical sample with trauma symptomatology. The goal was to recruit at least five and up to 15 participants, ensuring age and gender representation. Research has shown that gender plays a role in usability testing with children and young people, with girls tending to report more issues than boys [28]. Studies indicate that testing with 5 participants per target group is sufficient to identify approximately 80% of usability problems [29].

The recruitment process consisted of 2 steps. First, the coresearchers identified other young people within their networks, provided initial information, and inquired whether they might be interested in participating in the study. These young people were primarily from the coresearchers’ school networks and, like the coresearchers, resided in 1 large city and 1 medium-sized city in southern Sweden. If interested, they were then contacted by the academic researchers, who provided additional oral and written information and obtained consent for participation. The date and time of each usability test were mutually decided, with most sessions taking place in the evenings, as the coresearcher, participant, and researcher all needed to be physically present on-site. Five of the coresearchers conducted individual usability tests with participants (n=11; 6 male and 5 female; 15-20 years) over a 6-week period in January and February 2022.

#### Usability Testing Methods

To assess the app’s usability, the think-aloud method was applied for data collection. This method involves participants verbalizing their thoughts while completing specific or nonspecific tasks. Participants are encouraged to articulate what they are looking at, thinking, doing, and feeling as they navigate the app. During the usability test, the observer records participants’ verbalized thoughts and actions without interpreting them, paying particular attention to moments or areas where users experience difficulties [30].

All usability tests were conducted on university premises, with the coresearcher and participant in the same room and an academic researcher in an adjacent room. The participant was provided with a mobile phone and instructed to navigate through the entire app using the main menu while thinking aloud. The coresearcher documented the participant’s verbalized thoughts and observed behaviors as described above. After completing the app test, participants were asked by the coresearcher to complete the System Usability Scale (SUS) [31], which had been translated into Swedish. Following this, they participated in a structured interview led by the coresearcher to capture their general impressions of the app [32]. All notes from the test sessions were documented in Research Electronic Data Capture (REDCap), a secure online survey platform, during the session. No voice or video recordings were made.

#### Usability Testing Analysis

It was agreed in advance that immediately after each session, the coresearcher would report any major or serious usability problems identified during testing to the academic researcher. Following the procedure described by Hertzum [32], 2 academic researchers independently reviewed all notes from the test sessions, each compiling a list of positive and negative usability issues. These lists were then compared, discussed, and merged into a single final list. This consolidated list of usability issues was presented to the coresearchers in a dedicated meeting (Figure 2). Using a live interaction polling tool [33], the usability issues were displayed, and coresearchers individually rated their severity and suggested a priority order. The final prioritization was determined through group discussion.

### Ethical Considerations

The coresearchers participated in the project as experts rather than as participants of the study; therefore, standard ethical requirements did not apply. However, their safety was carefully considered throughout the process. From the outset, it was made clear that coresearchers were not expected to share personal experiences of trauma, as this was not necessary for the project. Discussing one’s own traumatic experiences could lead to retraumatization or secondary traumatization of others in the group [34]. To mitigate this risk, case vignettes were used in the workshops when developing app content related to trauma. This approach enabled coresearchers to engage in discussions about different traumatic experiences in a general manner rather than on a personal level.

Another key safety consideration was the nature of the tasks assigned to coresearchers between workshops. To minimize the risk of any negative impact, tasks conducted outside the workshop setting focused on app design and layout rather than potentially sensitive content.

The usability testing phase, involving young people as study participants, was submitted to and approved by the Swedish Ethical Review Authority (reference number 2022-04217-01). Study participants were fully informed about the research, and their consent was documented. As participants had access to the app’s full content during testing, a safety protocol was implemented. Before each test session, the academic researcher checked in with participants to assess their well-being. If a participant appeared unwell, the test session was not conducted. After each session, the academic researcher conducted separate follow-up conversations with both the participant and the coresearcher to ensure that no one had been adversely affected by the testing experience. If any issues arose during a session, such as a participant showing signs of distress, the coresearcher conducting the test would have immediately alerted the researcher, as practiced during the training session. As part of the safety protocol, the researcher documented the contact details of guardians for participants under 18 years of age before each test session.

## Results

### Phase 1: App Development

#### Overview

The TRT app was developed as a progressive web app using a no-code, cross-platform application development program [24]. During the initial discussions about compiling TRT into an app, the young people highlighted potential issues with native apps, particularly regarding data storage requirements. By building the app as a progressive web app, these concerns were mitigated while still providing a user experience comparable to that of a native app.

**Figure 3:**
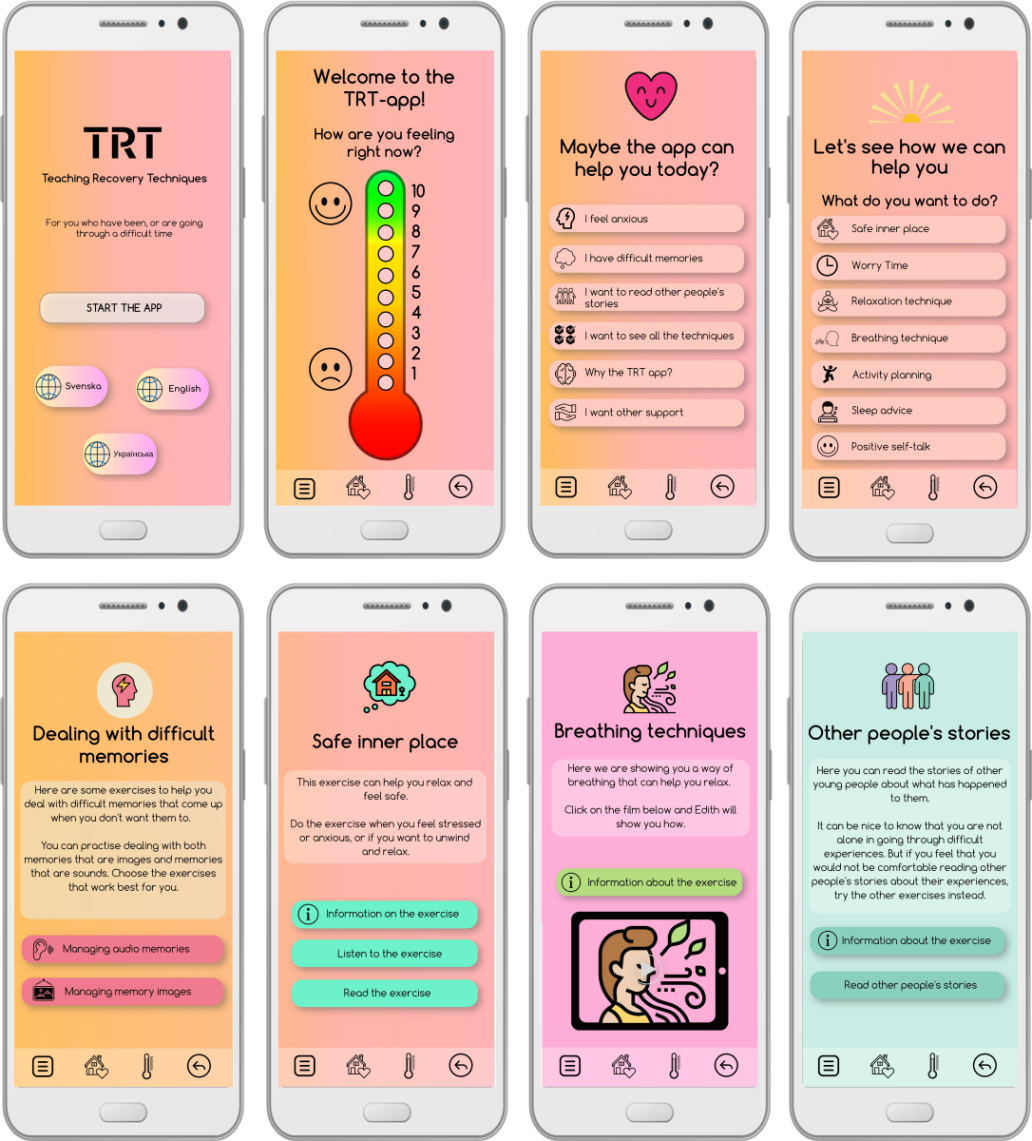
A selection of screenshots from the Teaching Recovery Techniques (TRT) app.

The coresearchers’ contributions to the app’s design can be categorized into 3 areas using the Mechanics, Dynamics, and Aesthetics (MDA) framework [35]. Mechanics refers to the fundamental rules and interactions that define the app’s structure. Dynamics encompasses what the user experiences in real time, such as the response when a button is pressed, which directly supports the app’s aesthetics. Aesthetics pertains to the intended emotional responses elicited in users when interacting with the app and includes its purpose and narrative. Beyond influencing visual elements such as colors, fonts, and symbols, the coresearchers placed significant emphasis on the overall user experience. A selection of app screenshots is presented in Figure 3.

#### Mechanics

The coresearchers’ contribution to the app’s mechanics—its basic rules and structure—included selecting techniques from the TRT manual, determining the format of the exercises, and structuring the app’s content. They emphasized the importance of the app’s structure, particularly the role of the main menu, which is the first thing users see upon entering the app. A clear and intuitive menu ensures easy and efficient navigation, allowing users to quickly find what they need. This not only saves time but also minimizes frustration when searching for specific features or content. The menu provides an overview of the app’s content, enhancing the user experience and improving usability. By featuring a front page with a well-organized menu, users can easily select their area of interest—whether it be reading texts, watching films, or completing exercises.

Both coresearchers with prior experience of TRT and those without agreed that the techniques in the app should follow the same order as in the manual—that is, as they would be introduced in a TRT group. There was consensus that the technique groupings should be labeled according to how young people who have had difficult experiences might feel (eg, “I feel anxious” or “I have intrusive memories”; Figure 3), making navigation more intuitive. Throughout the development process, coresearchers and academic researchers discussed the differences between being introduced to the techniques via an app versus in a facilitated group session. In the latter, group leaders present the exercises and provide support in response to participants’ reactions. Given this distinction, the coresearchers emphasized the importance of clear presentation within the app, ensuring that each exercise included information on its purpose and how to use it effectively.

#### Dynamics

The coresearchers emphasized the importance of the app being adaptable to the preferences of the young person using it, which is central to the app’s dynamics—the visible elements and user interactions. The content primarily consists of simple illustrative icons, instructional and demonstrational films, audio recordings, and short texts. Long texts were seen as detrimental to the user experience; however, careful consideration was required to shorten the content while maintaining fidelity to the TRT manual and the therapeutic intent of each technique. The descriptive texts introducing the techniques were kept short and simple. For young people seeking more information about the purpose of each technique, an “Information about the exercise” option is available (Figure 3). To enhance customization, most exercises offer the choice of either reading or listening to the instructions. Additionally, exercises presented as videos include optional subtitles.

#### Aesthetics

The app’s visual aesthetics were intentionally kept simple. The screen layout was designed to be uniform, featuring a representative icon and header at the top, followed by text or buttons framed by softly rounded boxes. An “easy-to-read” font with rounded edges was selected. The orange-pink color of the logo was chosen for its soft and friendly appearance—described as being “like the sun coming in from the left.” The app’s color theme was primarily based on the logo’s colors, with additional shades introduced throughout development to enhance variation and create clearer visual distinctions between different techniques (Figure 3).

The app’s aesthetics also relate to the emotional responses it aimed to evoke in users. The coresearchers emphasized that in-app films and voice recordings can be particularly helpful in promoting a sense of calm. These elements were seen as valuable for creating a relaxed atmosphere, providing guidance and instructions, and adding variety and creativity to the app experience. At the same time, the coresearchers stressed the importance of offering different exercise formats, recognizing that individuals have diverse preferences and needs when it comes to relaxation. While some may prefer breathing exercises, others might find meditation or guided visualization more effective. By providing a wide range of exercises, the app can better accommodate different users. In the videos, exercises are led by young people, as the coresearchers underscored the importance of featuring individuals with whom they can identify or relate.

### Overview of App Content and Navigation

#### Basis of Support Techniques

The trauma support techniques featured in the app are based on the TRT manual and incorporate several common approaches to trauma intervention, including psychoeducation, normalization, relaxation, and cognitive shifting. Psychoeducation provides individuals with information to enhance their understanding of psychological conditions—PTSD in this context. Normalization helps users recognize that their symptoms are not signs of weakness or character flaws but rather a natural response to their psychological condition. Relaxation techniques aim to alleviate stress-related symptoms, while cognitive shifting involves deliberately redirecting one’s attention from a singular fixation to alternative focal points. For safety reasons, the TRT app intentionally excludes in vivo exposure—a technique that involves directly confronting a feared object, situation, or activity in real life. A comprehensive overview of the app’s content is provided below.

#### Main Menu

To facilitate navigation for both new and experienced users, the app offers 2 ways to explore its content. In the main menu, the “Why the TRT app?” option provides first-time users with an introduction to TRT, trauma, trauma reactions, and how the app is intended to be used. Additionally, the main menu is structured around the user’s immediate needs, suggesting groups of exercises based on whether they are feeling anxious, experiencing intrusive memories, or seeking to learn more about trauma through psychoeducation and normalization of trauma reactions. For young people who are more experienced with TRT or the app and want to find a specific exercise, the main menu includes the option “I want to see all techniques.” This section lists all exercises in the order they appear in the TRT manual, allowing users to either select an individual exercise or follow a sequence similar to that of a facilitated TRT group. Additionally, the main menu features the option “I want other support,” which provides a comprehensive list of care and support organizations, including phone numbers and links to relevant web pages.

#### Thermometer

The Thermometer is the first screen displayed in the app, prompting the young person to rate how they are feeling on a scale from 1 (low mood) to 10 (high mood) before accessing the app’s content. If they report a score of 3 or lower, they are directed to a screen with instructions to call the national emergency number or contact a suicide prevention line if they are experiencing thoughts of self-harm. From there, they can choose to either proceed directly to the main menu or visit the “Other support” screen, which provides a list of care and support organizations. If a score above 3 is reported, the young person is encouraged to return to the Thermometer if their mood changes while using the app, before being directed to the main menu. The Thermometer remains accessible from every screen in the app via a tab bar (described below).

#### Safety Features

Techniques from the TRT manual that involve in vivo exposure or pose a risk of causing high levels of arousal or negative emotions have been excluded from the app or modified to minimize these risks. Techniques in the app that still involve recalling traumatic memories include shortcuts to safety features. Young people who feel they need additional support beyond what the app provides can access the “Other support” screen from the main menu or the “Thermometer” if they score low. This screen offers an extensive list of links and contact details for various digital and in-person care and support services for young people, providing more personalized help. The app only lists these services; no referrals are made.

The “Safe Inner Place” technique helps young people take control of intrusive memories and relax their bodies during periods of high arousal. This visual imagery technique allows users to create positive mental images, enabling them to influence their imagination and emotions. It offers 2 options: reading or listening to the guide. The Safe Inner Place serves as a secure base that users can return to when experiencing high arousal. Easy access to this feature from the tab bar (described below) was seen as empowering, allowing young people to explore more challenging exercises with greater confidence.

#### Tab Bar

A tab bar with 4 icons is located at the bottom of each screen in the app. These icons provide quick access to the Main Menu, the Safe Inner Place technique, the Thermometer, and the previous screen. The tab bar is designed to facilitate navigation while also serving as a safety feature. If a young person experiences high physiological arousal or negative emotions while using the app, they can quickly access the Safe Inner Place relaxation technique or the Thermometer. If they indicate a low level of well-being, the Thermometer will present them with options for additional care and support.

#### Anxiety Reducing Exercises

From the main menu, selecting “I feel anxious” directs the young person to a submenu of techniques designed to help manage negative emotions related to trauma. In addition to the Safe Inner Place technique, 6 other techniques support emotional regulation and coping with arousal and intrusive thoughts. Among them, 2 video-based exercises—*Breathing Technique* and *Relaxation Exercise*—are specifically designed to promote relaxation and help the young person regulate physiological arousal.

*Activity Scheduling* is a text-based technique designed to encourage social and physical activity while providing a distraction from anxious thoughts and feelings. The exercise offers 3 options: receiving suggestions for meaningful activities, creating a personalized list of activities, or scheduling planned activities. The suggested activities are provided by youth advisors to ensure they are relevant to young people. Another text-based technique, *Worry Time*, encourages setting aside 10 minutes each day for intrusive thoughts and worries, helping to limit worry time and regain control over intrusive thoughts. *Sleep Advice* provides a list of tips to improve sleep hygiene for young people experiencing sleep difficulties or nightmares due to trauma. Finally, *Positive Self-Talk* helps young people recognize the connection between thoughts and feelings and learn to replace negative self-perceptions with positive ones. The exercise offers a choice between receiving suggested positive affirmations or creating personalized ones.

#### Techniques for Intrusion Control

Selecting the option “I have intrusive memories” from the main menu provides a choice of techniques to help manage auditory or visual intrusive memories. *Coping with Sound Memories* is a video-based exercise where the young person practices controlling intrusive auditory memories by mentally manipulating the sound in different ways. Coping with Visual Memories includes 2 exercises: *Screen Techniques* and *Clapping Technique.* Screen Techniques is a video-based exercise that, similar to the Auditory Intrusion Exercise, guides users in manipulating visual memories as if they were displayed on a TV screen or in the palm of their hand to regain control over them. The Clapping Technique, known as bilateral stimulation in the TRT manual—a term the coresearchers found too complex for the app—demonstrates how rhythmically tapping the knees while recalling intrusive images can help modify and regulate those memories.

#### Other People’s Stories

The “Other People’s Stories” page is accessible via a button in the main menu. It features a collection of fictional stories in which young people describe their personal experiences of trauma in writing. The purpose of these stories is to provide psychoeducation and normalize emotional and psychological reactions to trauma. The traumatic events depicted were suggested by the coresearchers, with plans to expand the content to include a more diverse range of experiences. At the time of usability testing, the available stories covered themes such as losing a parent, being bullied, and experiencing war. Each story is clearly labeled with a heading that indicates its subject matter.

### Phase 2: Usability Testing

For the SUS, complete data were available from 10 participants, with scores ranging from 67.5 to 97.5 [32]. Based on existing reference values, a product must achieve an SUS score of at least 71 to be in the top half [32,36]. Nine out of the 10 completed SUS recordings had scores of 77.5 or higher.

Table 1 summarizes the identified usability issues along with the corresponding modifications. Some issues were related to technical preferences, such as the ability to rewind video and audio files to revisit missed content or replay important segments. Additionally, the option to enable subtitles was considered important for those who wished to use them. As a result, rewind and subtitle features were added to all audio and video recordings. While the video format was well received, some recordings were noted to have low quality, with slight flickering from background lighting. Some participants reported that this negatively impacted the overall user experience. To address this, all relevant videos were rerecorded in a professional studio, with the same young person demonstrating the techniques.

**Table 1:** Usability issues identified with the prototype of the Teaching Recovery Techniques app and the corresponding modifications.

Usability issues	Modifications to the app
Some of the video and audio recordings lacked the ability to rewind as well as the option for subtitles.	Rewind function and option for subtitles were added to all videos and recordings.
Users found the quality of some video and audio recordings to be low, affecting the overall user experience negatively.	The videos were rerecorded in a studio. The audios were rerecorded by the same narrator, while ensuring no interferences in the recording.
Users were bothered by difficulties related to the device, such as the screen going black during long audio and video recordings, and the lower tab bar being difficult to access with an iPhone.	Instructional text was added, directing the user to the device settings to enable the screen to stay on for an extended period. Adjustments were made so access to the tab bar is not affected by the type of device.
The *Other People’s Stories* exercise, aimed at identification and normalization, was generally perceived as important and helpful, but the audio recordings were also perceived as stressful to listen to. It could also be perceived as pressurizing to hear from others who have managed to overcome their difficulties.	A decision was made to keep *Other People’s Stories* in text format but exclude the audio recordings from the app. Additional information about the contents of the stories, as well as the purpose of the exercise, was added. A reference to other exercises in the app in case the user feels they would not benefit from reading other people’s stories was also added.
Users reacted to the advice to “reach out to someone close to you” and expressed that some people don’t have that option, which may be why they use the app.	A reference to the *Other Support* screen was added.
There was too little variety in the tips on sleep, activities, and positive self-talk.	More tips were added for each exercise.
The app only being available in Swedish.	The app was translated into more languages.

Usability issues related to device functionality were also identified. Some video and audio recordings lasted several minutes, and if participants remained passive during playback—without touching the screen—the display would time out, disrupting their ability to focus on the exercise. To address this, a reference was added to each technique’s information, guiding users on how to adjust screen time-out settings on their devices. Another device-related issue involved the tab bar at the bottom of each screen, which was unnecessarily difficult to access on iPhones. Adjustments were made to ensure a consistent user experience of the tab bar across different devices.

Some aspects were identified as potentially incompatible with the reality of the target group. Participants responded to the recurring advice to “talk to a trusted person,” noting that some users might not have this option—which may, in fact, be why they turn to the app. To address this, a clarification was added, stating that if the user cannot or does not want to talk to someone close to them, the “Other support” section in the app provides several recommendations for both in-person and digital support services.

Additionally, the option to listen to other people’s stories, narrated by young people for identification purposes, was found to carry the risk of causing undesirable feelings. Hearing someone speak about their personal difficulties could be burdensome and might also create pressure for the listener to cope with their own situation. As a result, the audio option was removed, leaving only the text format. Additional information was added to clarify the content and purpose of the stories. A reference to other exercises in the app was also included, along with the following message: “If you feel that reading other people’s stories may not be helpful for you, you can try these exercises instead.”

## Discussion

### Principal Findings

This project was conducted with young people who had personal experiences of trauma, actively involving them as experts from an early stage. In line with the Lundy model, creating a safe and inclusive space for children to express their views proved essential from the outset and remained a priority throughout [17]. All activities, except for the usability tests, were held in a conference room at a centrally located hotel. The coresearchers expressed early on that they appreciated the setting, describing the space as cozy, the food and refreshments as enjoyable, and the environment as one where they felt comfortable expressing themselves.

Careful consideration was given to recruitment strategies to ensure that young people from diverse backgrounds and experiences were informed about the project. While previous participatory research projects have highlighted the challenges of recruiting young people [37], our experience was different, with several young individuals quickly expressing interest. The use of multiple recruitment arenas proved valuable, and further exploration of effective strategies for engaging young coresearchers is warranted.

With regard to voice [17], coresearchers were encouraged to express themselves in various formats. Some felt comfortable speaking freely in workshops, while others preferred responding to direct questions. One coresearcher expressed early on that writing down their thoughts during workshops would be easier than speaking in a group. This option was made available and encouraged for all participants. Even those who preferred verbal communication occasionally utilized written expression. A standing agreement ensured that coresearchers could share their thoughts through writing, drawings, or digital images, sometimes compiled into vision boards. Several coresearchers contributed their insights this way at least once during or after a workshop. Likewise, both written and verbal information was consistently provided to accommodate different preferences.

Research has shown that participatory approaches with young people must be flexible and pragmatic, aligning with their preferences and opportunities. Participation should be offered at different levels and in various formats, allowing young people to decide how they engage [38]. In this project, coresearchers’ preferences guided practical aspects such as venue selection, scheduling, and breaks, including refreshments (see Sarkadi et al [39] for more details). All workshops were held in the same easily accessible venue, ensuring convenience for participants using any mode of transport. To foster a safe and inclusive environment, coresearchers collaboratively developed guidelines outlining how they wished to work together, including common rules for respect and understanding. A key expectation, as expressed in these discussions, was that open conversations would be easier if participants were assured that no one would interrupt or dismiss their opinions.

Sufficient time was allocated to allow researchers to work cocreatively with the coresearchers, ensuring the flexibility required for this type of collaboration. Joint workshops were scheduled in the evenings and on weekends, allowing coresearchers to participate without missing school—an aspect they highlighted as important. Additionally, researchers remained accessible throughout the project via multiple communication channels, accommodating individual preferences. Early in the project, a discussion was held to establish mutually agreed-upon times for communication. The coresearchers generally preferred communicating in the evenings but understood that responses might sometimes take until the next day. This flexibility contributed to consistently high workshop attendance. When a coresearcher was unable to attend, they were offered an individual meeting or conversation with a researcher, as they expressed a strong interest in staying engaged with the project’s progress.

While enabling expression is important, so is providing the option not to participate. Early in the project, the coresearchers emphasized that they sometimes have a lot going on in their lives, particularly with school. To minimize absences, proposed dates and times were communicated in advance, allowing for joint decisions on scheduling. Additionally, the youth advisors were reminded that they could choose which parts of the process they wanted—and were able—to be involved in.

Audience refers to whether and how young people’s views are being listened to [17]. Each workshop was attended by a researcher (MT) and the research assistant responsible for app development (KE), ensuring a direct communication pathway from coresearcher idea generation to app implementation. Having 2 adults present also allowed for more effective engagement—while one (KE) took notes, the other (MT) could focus on facilitating discussions and actively listening. To demonstrate that their input was valued, ideas from previous workshops and independent work tasks were shared back with the coresearchers at each session. This process was further reinforced by sharing app prototypes developed based on decisions made in previous workshops. This aligns with the Influence component of the Lundy model [17], as it allows youth advisors to see their input being implemented in real time. Additionally, the project was presented at multiple scientific conferences and regularly reported to the funding organization. Preparations for these events, as well as feedback received, were always shared with the coresearchers to ensure transparency about how and where their contributions were being presented. Those who wished to be recognized were also listed as coauthors on conference poster presentations.

The influence of the coresearchers extended beyond app development to the usability testing process. When reflecting on their experience, they described it as “fun and easy to do.” They emphasized the value of peer-led research, noting that having young people conduct the testing and recruit from their own friendship networks made participants more comfortable expressing their true opinions—both positive and negative. By contrast, they felt that if adult researchers had conducted the testing, participants might not have responded as openly. The coresearchers emphasized that their close age and shared experiences with the participants allowed them to relate more directly, fostering a sense of understanding and connection. As peers, they created a more relaxed and informal atmosphere, making it easier for participants to communicate openly and feel comfortable. These reflections align with existing literature, which suggests that having observers and users with similar backgrounds can reduce misinterpretation of data in usability testing [32]. Arguments have been made in favor of involving children and young people as coresearchers, as their firsthand experience of childhood provides an insider perspective and expertise that adults cannot fully replicate [16,40]. While adult researchers may be considered outsiders, young people are embedded in peer culture, allowing them to be closer to the subject of research [41]. In line with the coresearchers’ reflections, peer-led data collection has been shown to generate nuanced and valuable insights that differ from those typically produced by adult researchers [41].

### Methodological Considerations

This study took steps to ensure diversity among coresearchers, representing different genders, ages, and backgrounds. However, it cannot be assumed that they fully represent all young people who have experienced trauma. The recruitment of study participants by the coresearchers is largely a strength, as their diversity was reflected in the sample. One limitation of the study was the decision not to video record the usability testing sessions, which could have provided additional insights into participant behavior. However, combining a standardized instrument with interview questions after usability tests was a strength, as was the multistep analysis process, which involved 2 researchers when applicable.

### Conclusions

This study demonstrates that young people with personal experiences of trauma can actively contribute as coresearchers in the development and usability testing of a trauma support app. A child rights framework can facilitate this process, ensuring alignment with Article 12 of the CRC. The young people’s contributions to the app’s design can be categorized into 3 key areas: mechanics, dynamics, and aesthetics. The design studio method proved to be an effective approach for cocreation, allowing for both the generation and refinement of their ideas. No formal training was required for the young people to contribute as experts in the development process. However, relevant training was essential to equip them for independent data collection. In parallel with this project, work has been conducted on potential negative consequences of the app—referred to as dark logic—which has been reported elsewhere [42]. The next phase involves further testing the app in a larger population. Ongoing efforts are focused on piloting the app both with young people participating in a TRT group and as a stand-alone intervention.

## Abbreviations

CRC: the Convention on the Rights of the Child
MDA: Mechanics, Dynamics, and Aesthetics
PTSD: posttraumatic stress disorder
REDCap: Research Electronic Data Capture
SUS: System Usability Scale
TRT: Teaching Recovery Techniques

## Data Availability

Data generated for this study are not publicly available to protect the privacy of those involved. Requests to access the data should be directed to GW.
